# Barriers and facilitators for adolescent girls to take on adult responsibility for dental care – a qualitative study

**DOI:** 10.1080/17482631.2019.1678971

**Published:** 2019-10-13

**Authors:** Anida Fägerstad, Jesper Lundgren, Kristina Arnrup, Eva Carlson

**Affiliations:** a Public Dental Service, Region Örebro County, Örebro, Sweden; b Faculty of Medicine and Health, School of Health Sciences, Örebro University, Örebro, Sweden; c Department of Psychology, University of Gothenburg, Gothenburg, Sweden; d Faculty of Medicine and Health, University Health Care Research Center, Örebro University, Örebro, Sweden

**Keywords:** Dental care, dental attendance, adolescent, content analysis

## Abstract

**Purpose**: This study aims to explore and describe experiences of the dental care system among adolescent dental patients with a recent history of missed dental appointments at public dental clinics (PDCs) in a Swedish county.

**Methods**: Twelve adolescent girls participated in the study. Data were collected by individual, semi-structured, open-ended interviews and analysed with qualitative content analysis.

**Results**: The study findings could be summed into the theme “Triggers for adolescent girls to take on or not take on adult responsibility for dental care”. The experience of free dental care could be summarized in five main categories: Pain and discomfort; Attractive and healthy teeth; Feeling safe and secure; Taking on the responsibility; and Free of charge. These five categories consisted of 15 subcategories.

**Conclusions**: The results of this study should increase the knowledge on how to meet and treat adolescent girls in dental care. Knowing what will happen during the dental visit was highlighted by the participants as decisive to whether or not they would attend their dental appointments. Therefore, we should as far as possible ensure that our patients feel safe at their dental visits and by trying to avoid painful treatments.

## Introduction

Dental health services differ between countries in terms of organization, accessibility, availability and cost (World Health Organization, 2015). In some countries, full dental health services are readily available through private or public systems, while in other countries, dental services are limited to pain relief and emergency care (Socialstyrelsen, 2017). To ensure access to dental care regardless of socioeconomic or insurance status, all children and adolescents living in Sweden are offered free dental care with regular check-ups at intervals determined by individual risk assessments (Socialstyrelsen, 2015).

Adolescence is a period of physical, psychological, sociocultural and cognitive development and a period of transition from childhood to adulthood (Rivara, Park, & Irwin, 2009). The goal of many adolescents is to be free from their parents and to have control over their own lives (Crosby, DiClemente, & Santelli, 2009). Even though they want to be independent, most adolescents still want to have a close relationship with their parents (Hwang, 2018). The entire adolescence may feel like a balance between independence and dependence from family, peers, and the community (Christie & Viner, 2005). The relationship with peers and social responsibility become more central, while the relationship with family becomes less prominent (Crosby et al., 2009). Further, the opinions of peers become more important than opinions from the family (Blakemore & Mills, 2014). During this period, adolescents may establish health behaviours that can affect health through their whole life (Rivara et al., 2009).

Oral health behaviours such as tooth-brushing habits, use of fluoridated toothpaste, and sugar consumption have been found to have an effect on oral health status (Gherunpong, Tsakos, & Sheiham, 2006). Broken appointments and irregular dental attendance have been associated with negative oral health effects as well as emergency care (Armfield, 2013; Crocombe et al., 2011; Klingberg, Berggren, & Noren, 1994; Wang & Aspelund, 2009; Wigen, Skaret, & Wang, 2009; Wogelius, Poulsen, & Sorensen, 2003). In order to keep good oral health through the life course, individuals ought to maintain an individualized but regular attendance pattern in the dental health care system.

Despite free dental care for children and adolescents living in Sweden, reports on missed and cancelled dental appointments are not unusual. For example, 8% of children and adolescents in one Swedish county missed their dental appointments in 2005 (Nordenram, 2012). Of those individuals, the largest proportion, of 11%, was found among 19-year-olds. The same report revealed that many of those 19-year-olds would only visit a dentist because of pain or other problems (Nordenram, 2012). Another study (Östberg, Ericsson, Wennstrom, & Abrahamsson, 2010) showed that approximately 35% (30% of girls and 41% of boys) of 19-year-olds in another Swedish county did not plan for future regular dental visits when they needed to pay for their dental care.

Missed and cancelled dental appointments in the context of free dental care have been associated with a variety of background and concomitant factors (Fägerstad, Windahl, & Arnrup, 2016). For example, it has been shown that dental avoidance is more common among boys (Östberg et al., 2010; Skaret, Berg, Kvale, & Raadal, 2007; Skaret, Raadal, Kvale, & Berg, 1998, 2000) and older adolescents (16**–**18- year-olds) (Skaret et al., 1998). Further, irregular dental care was more common among adolescents attending a vocational upper secondary programme than among those following an academic upper secondary programme (Ericsson et al., 2016). A recently published study based on dental records revealed that adolescents with missed dental appointments had more oral health problems, more invasive dental treatments and, in the past, more missed and cancelled dental appointments (Fägerstad, Lundgren, Windahl, & Arnrup, 2018). Furthermore, pain experience during dental treatment (Skaret, Raadal, Berg, & Kvale, 1999; Vika, Raadal, Skaret, & Kvale, 2006), unpleasant dental visits (Ekman, 1989; Östberg, Halling, & Lindblad, 1999) and giving low priority to dental care (Östberg et al., 2010; Skaret et al., 2000) have been reported as other factors associated with irregular dental attendance among adolescents.

Despite all these quantitative studies on dental avoidance among adolescents, we only found one qualitative study focusing on dental avoidance, conducted with parents of children and adolescents with irregular dental attendance. The study revealed that parents who felt overloaded in daily life did not prioritize taking their children for dental care. Moreover, these parents themselves were irregular dental attenders and gave low priority to their own oral health (Hallberg, Camling, Zickert, Robertson, & Berggren, 2008). This indicates that missed dental appointments among adolescents in a system of free dental care still remain a challenge.

As far as we know, there are no studies on how adolescents who miss their dental appointments experience Swedish dental care. In order to find ways to prevent irregular dental attendance, there is a need for in-depth knowledge about adolescents’ experiences of the free dental care service. Therefore, the aim of this study was to explore and describe experiences of the dental care system among adolescent dental patients with a recent history of missed dental appointments at public dental clinics (PDCs) in a Swedish county.

## Method

### Design

This study has an inductive, descriptive design with a qualitative approach (Elo & Kyngäs, 2008; Graneheim & Lundman, 2004). Data were collected by individual, semi-structured, open-ended interviews and analysed with qualitative content analysis according to Graneheim & Lundman (Graneheim & Lundman, 2004).

### Participants

The study was performed in 16**–**19-year-old dental patients with missed dental appointments at any of the PDCs (n = 24) in Örebro County, Sweden, during the preceding 3 months in 2018. Notes on missed appointments during the study period were regularly reported to the first author by a controller, prior to individual contact.

Eligible participants were purposefully selected aiming for diversity of age, gender and PDCs’ location in areas with different sociodemographic profiles (urban, small towns, rural, low and average/high socioeconomic status (SES)) to find a variety of ways of experiencing the phenomenon we wanted to study.

### Ethical considerations

This study was approved by the Regional Ethical Review Board in Uppsala, Sweden (reference number 2017/281), and was performed in accordance with the principles stated in the Declaration of Helsinki (Association WM, 2017). Since all adolescents were between 16 and 19 years of age, no parental consent was needed for participation, in accordance with the ethical vetting legislation in Sweden (SFS 2003:460, §18). The adolescents received both written and verbal information about the study, and were informed that their participation was voluntary and that they could withdraw from the study at any time, without giving any reason, and without consequences for their future dental care. Written informed consent was obtained.

### Procedure

At first contact, the eligible participants were sent an information letter with a short description of the study aim and procedure. The letter further contained a declaration that participation was voluntary and that data collection, analysis, reporting and data storage would be treated confidentially. About 1 week after the letter was sent, the adolescents were contacted by phone by the first author (A.F.) and asked whether they were interested in participating in the study. They were also given the opportunity to ask questions about the study.

In total, 2,335 adolescents missed their dental appointments during the period from January to September 2018. Of those adolescents, the information letter was sent to 152 individuals during that period. Telephone contact was established with only 18 (one boy and 17 girls) out of the 152 individuals, while the others were not reachable. Four individuals, all girls, chose not to participate in the study giving reasons such as no interest or being on vacation. The boy and one girl agreed to participate but did not show up at the interview session. Therefore, no boys participated in the study. In total, twelve girls agreed to participate (Table I). Because of difficulties in finding time for the interviews, some of the individuals stated that they preferred a telephone interview over a face-to-face interview. Thus, an appointment was arranged for face-to-face interviews with eight participants while four participants were interviewed by telephone. The interviews were conducted in Swedish.

**Table I.** Participants’ characteristics and demographics.

Participants	Age, yrs	Type of interview	PDCs’ location and sociodemographic profile of the area	
1	19	Face-to-face	Small town	Average/high SES
2	19	Face-to-face	Rural	Average/high SES
3	16	Face-to-face	Urban	Low SES
4	18	Face-to-face	Urban	Average/high SES
5	18	Face-to-face	Urban	Average/high SES
6	19	Face-to-face	Urban	Average/high SES
7	17	Face-to-face	Urban	Average/high SES
8	18	Face-to-face	Small town	Average/high SES
9	19	Telephone	Urban	Average/high SES
10	18	Telephone	Small town	Average/high SES
11	19	Telephone	Urban	Average/high SES
12	19	Telephone	Rural	Average/high SES

PDC = public dental clinic; SES = socioeconomic status.

### Data collection

Data were collected between February and September 2018 through twelve individual, open-ended semi-structured interviews by using an interview guide based on earlier research (Dodd, Logan, Brown, Calderon, & Catalanotto, 2014; Ericsson, Östberg, Wennstrom, & Abrahamsson, 2012; Östberg et al., 2010) and the researchers’ clinical experiences. The interview started with questions about the participant’s last dental visit and latest missed dental appointment. The questions in the interview guide focused on missed dental appointments, barriers and facilitators to accessing dental care, attitudes to oral health, and peer and parental influence on dental attendance. Probing and follow-up questions were asked, such as “Can you explain more?”, “Can you tell me more about it?”, “Can you give an example?”

The first author, who is a registered dental hygienist, carried out all the interviews. None of the participants had had any previous contact with the interviewer (i.e., was not treated by her as dental hygienist at any time).

In order to practise qualitative interviewing and test the relevance and the construct validity of the interview guide regarding the study aim, the first author conducted five pilot interviews. They resulted in some minor changes made to the interview guide. None of the pilot interviews were included in the study because the individuals did not meet the inclusion criteria.

Face-to-face interviews were conducted in a quiet room at the research centre and lasted between 12 and 38 minutes (mean 23 minutes). Time for the telephone interviews ranged from 14 to 19 minutes (mean 16 minutes). Before an interview started, the interviewer once again explained the aim of the study and informed the participant that the interview would be recorded digitally. The participants were free to express themselves when answering the open-ended questions about their experiences of dental care. All interviews were digitally recorded and transcribed verbatim. The first author transcribed the first interview while two experienced secretaries transcribed the rest of the interviews shortly after they were conducted. The transcripts were checked and, if necessary, corrected by the first author by listening to the audio files.

After having conducted all the interviews, the first author together with co-authors E.C. and K.A. checked whether any new topics had emerged during the last interviews, or whether no additional information could be found.

### Data analysis

The data were analysed using qualitative content analysis with an inductive approach guided by Graneheim & Lundman (Graneheim & Lundman, 2004). The method focuses on subject and context and emphasizes similarities within and differences between parts of the text (Graneheim, Lindgren, & Lundman, 2017; Graneheim & Lundman, 2004).

Considering the fact that only girls agreed to participate and therefore that the heterogeneity of the group of participants was limited, no new information related to the studied phenomenon was obtained in the last four interviews. Therefore, it was decided that twelve interviews were enough to achieve the aim of the study.

Two authors (A.F. and E.C.) read through the interviews several times to gain a sense of the data before continuing with the analysis. Thereafter, the meaning units including statements relevant to the study aim were extracted from the transcripts. The meaning units were then condensed, abstracted and labelled with a code. The codes were compared for similarities and dissimilarities and then grouped together into main categories and subcategories, which constitute the manifest content. The preliminary subcategories and categories were discussed several times by two authors (A.F. and E.C.) and revised. The other co‐authors (K.A. and J.L.) had a validating role throughout the analysis process. In the last step, the underlying meaning with the latent content of the categories was formulated into a theme (Graneheim & Lundman, 2004).

## Results

The experience of dental care among adolescent girls with a recent history of missed appointments could be expressed as a theme, “Triggers for adolescent girls to take on or not take on adult responsibility for dental care”. The five categories Pain and discomfort; Attractive and healthy teeth; Feeling safe and secure; Taking on the responsibility; and Free of charge consisted of 15 subcategories (Table II).

**Table II.** Overview of the theme, categories and subcategories illustrating adolescent girls’ views on Swedish dental care.

Triggers for adolescent girls to take on or not take on adult responsibility for dental care				
Pain and discomfort	Attractive and healthy teeth	Feeling safe and secure	Taking on the responsibility	Free of charge
Effects of previous negative dental experiences	The importance of healthy teeth	Understanding what will happen at the dental clinic	To be reminded	Make sure to go for dental care while it’s free
Negative peer influence		Having someone accompany you to the dental clinic	Ambivalence about taking on the responsibility	Wouldn’t pay for it
Having dental instruments in the mouth		Having confidence in dental personnel	Prioritizing other things over dental care	
		Being criticized by dental personnel	Transportation difficulties	
			Difficulties getting in contact	

### Pain and discomfort

This category consisted of the three subcategories Effects of previous negative dental experiences; Negative peer influence; and Having dental instruments in the mouth. A general finding was that the participants described themselves as not afraid of the dentist, but being at the dental clinic was often described as an unpleasant experience. One of the instances of feeling discomfort at the dental clinic visit was when the participants had to open their mouths for the dental personnel. In addition, they described dental personnel putting at least three or four instruments and their fingers in their mouth during the examination and dental treatment. Such experiences created feelings of pain and discomfort.

#### Effects of previous negative dental experiences

The experience of local anaesthesia and having undergone operative dental treatment such as tooth extractions and fillings in the past was described as painful and unpleasant, or even dreadful, and affected the participants’ feelings about future dental visits. These negative dental experiences made them unsure about whether they would go for dental care in the future.

It was that time when a dental hygienist gave me local anaesthesia and my whole tongue was anaesthetized and then they booked me to visit her again, but I called and told them that I didn’t want to be treated by her again, but it was solved anyway. So if that would happen again I wouldn’t want to go to the dentist any more. (P 1)

#### Negative peer influence

Talking negatively about dental personnel was not unusual within the participants’ peer groups. The adolescents would frighten each other by talking about having sharp instruments in the mouth that could hurt them. In addition, they sometimes wondered if dental personnel would hurt them by using those sharp instruments during the dental treatment. Frightening each other could sometimes make the participants more afraid than they were before talking to their peers. However, they said that they would not miss their dental appointments because of what their peers said.

But there are some friends, when you tell them that you’re afraid of the dentist, they say, “I don’t like those sharp scalpels they stick in the mouth.”/ … /It’s more that you joke a little but you know that it [dental care] is important. You go [for dental care] anyway. Even though you are a little afraid. (P 5)

#### Having dental instruments in the mouth

According to the participants’ descriptions, having X-rays was one of the most painful procedures they needed to undergo when visiting the dental clinic. Being treated by a dentist who is rough or who makes rapid movements during the dental treatment was described as tough. Also, the participants expressed feelings of discomfort when they had to endure having different kinds of dental instruments in the mouth. They also described how they felt discomfort at having to have their mouth open for a long time. However, they saw this discomfort as something they needed to bear in order to ensure good oral hygiene.

That is something you have to do [enduring to have dental instruments in the mouth]. But, it’s like this, I hate having things in my mouth, like those steel instruments and I don’t know, and when you need to have your mouth open for so long … (P 5)

### Attractive and healthy teeth

The participants described an awareness that oral health could have an effect on health in general and that good oral health was a prerequisite to feeling good. They also described the importance of taking care of their oral health in the same way as taking care of the rest of their bodies.

#### The importance of healthy teeth

To have healthy teeth was important to the participants who said they wanted to keep their teeth healthy even in the distant future, when they got old. Also, having healthy teeth had a cosmetic aspect requiring white and clean teeth as well as being free of caries disease.

The appearance of healthy teeth was the highest concern when participating in social situations.

You want to smile. You don’t want to be ashamed of your teeth. So for me, it’s really important. No, I think oral health is important. You want to feel fresh and you don’t want to think about your breath when you talk to someone. (Participant (P) 4)

Going to the dental clinic was a way for participants to get an update about their oral health status. In other words, dental personnel provided them with information about presence of caries disease, and told them whether their oral health was good and whether their teeth were sufficiently clean. Further, the participants mentioned that they got instructions on how to take care of their teeth from the dental personnel.

I get a lot of help with how to take care of my teeth. Because I have very bad teeth. (P 8)

### Feeling safe and secure

Four subcategories formed this category, namely, Understanding what will happen at the dental clinic; Having someone accompany you to the dental clinic; Having confidence in dental personnel; and Being criticized by dental personnel.

#### Understanding what will happen at the dental clinic

Since dental visits are sometimes experienced as unpleasant, knowing what was going to happen during the visit was important for the participants to feel safe and secure. They did not only want to know what would happen during the present treatment; they also wanted to know what was planned for their next visit. Lack of this information could cause them not to go to their next appointment at the dental clinic.

I think it’s quite important because then you feel safe when you know what … Next time you come here you should do this. This time we should start with this. (P 12)

A spectrum of experiences of dental visits were described, with frightening as well as more neutral experiences. To tell the dental personnel about their feelings of fear was a successful strategy that also helped the personnel to make adjustments so that the visit could be more comfortable for the adolescent.

I think it’s a little bit tough with dental visits. I think. But it usually works out well, if you say that you are a little afraid, then it usually goes well. (P 6)

#### Having someone accompany you to the dental clinic

The participants recognized that they had the responsibility to manage their dental visits as they would soon be adults. However, having a parent or friend accompany them to the dental clinic was appreciated as it made them feel more safe and relaxed. If the participants felt awkward about talking to the dental personnel, their accompanying parent could be their spokesperson. On the other hand, if their parent was not able to accompany them to the dental clinic, adolescents themselves managed to speak out their opinions to the dental personnel if accompanied by one of their friends.

I think that it’s a comfort to have someone with you. It doesn’t need to be one of my parents; it can be someone else as well. Because it gets more relaxed in some way because you have someone there that you’re comfortable with./ … /When I have a friend, I can still feel that I can say that I’m a little nervous. (P 6)

#### Having confidence in dental personnel

A variety of preferences were described considering personal continuity of care. Generally, the participants preferred to have the same dental personnel treat them with whom they felt secure and they knew what to expect. Continuity helped them to go to the dental clinic. Also, the experience of meeting friendly dental personnel who could make them relax and feel calm was described as important. Moreover, the participants preferred dental personnel who would let them be involved, and not make them get stressed during the dental treatment. This helped them understand the purpose of their dental visits. Losing confidence in the dental personnel was described in the context of not being satisfied with the dental treatments or having no idea what would happen during the dental visit.

The dental hygienist or dental nurse can explain what they are doing so maybe I can get a deeper understanding of why I need to come to my booked appointments. (P 9)

#### Being criticized by dental personnel

The experience of feeling afraid of going to the dental clinic can sometimes be caused by criticism from the dental personnel. The participants described how dental personnel sometimes criticized them for not managing their dental hygiene and taking care of their oral health. Some participants even cancelled their dental appointments because they were afraid of being blamed for their bad dental hygiene.

I sometimes think it’s scary to go to the dental clinic because you can be blamed for not taking care of your teeth. (P 2)

Dental personnel were often described as strangers “doing things” in the participants’ mouth. Having dental personnel, someone they did not know, “hang over” their faces made them feel exposed and vulnerable. They also expressed loss of their integrity and privacy when dental personnel were very close to them.

Finally, the importance of hygiene, i.e., having clean instruments, meeting dental personnel who were clean as well as being treated at a clean dental clinic contributed to the participants’ descriptions of having confidence in dental personnel.

### Taking on the responsibility

The participants described varying degrees of independence and maturity. This category was made up by the five subcategories To be reminded; Ambivalence about taking on the responsibility; Prioritizing other things over dental care; Transportation difficulties; and Difficulties getting in contact.

#### To be reminded

The participants described that when they were small their parents had talked positively about the dentist and taught them about the importance of good oral hygiene. They learned from their parents that tooth brushing twice a day was important for having good oral health through the life course and they reported that their parents helped them brush their teeth when they were younger. The role of the parents was important even when it came to dental care visits.

The participants described that it could be difficult to remember the appointment time and talked about possible ways of being reminded about their dental visits. In most cases it was their parents who reminded them. When they missed their dental appointments, their parents made sure that they called the dental clinic to book a new appointment.

My [parents] have always reminded me and made sure that … Now that I have to pay … When I had missed [my dental appointment] my mum was very [on my case], saying, You need to book a new appointment. (P7)

Receiving short message service (SMS) text reminders from the dental clinic was another way for the adolescents to remember their appointments. When they missed an appointment, a dental nurse contacted them by telephone. The participants said they appreciated being contacted by the dental nurse as this gave them the opportunity to get another dental appointment.

#### Ambivalence about taking on the responsibility

The participants’ expressions of their personal ability and maturity to take on full responsibility for the dental visits ranged from stating that an adolescent should be responsible for their own dental visits and contacts with dental care, to saying that parents should still be responsible for the dental care of their adolescent and that the dental clinic should always contact the parents.

You have your own responsibility. You go to your booked appointments and if you can’t go, you should call and cancel or solve it in some way. (P 1)

The participants said that they would not pay for their dental appointments if they did not look after their teeth. Those who said they did not take care of their oral health, and that they disliked the dentist, were also more likely to reschedule or miss their dental appointments.

It may be that I know that I have been careless with the tooth brushing and therefore I have been rescheduling my dental appointments. (P 6)

#### Prioritizing other things over dental care

Low priority was given to the dental visits despite the importance of oral health that was recognized by the participants. If they had to choose between going to the dental clinic and doing something else, like being with friends, they would choose the latter. They would prioritize working, going to school or going to the doctor over going to the dental clinic. The participants also expressed that although they wanted to go to their dental appointments, they had trouble calling off activities together with their friends.

Maybe it’s uncool to say to your friends you can’t go with them and do things because you have a dentist appointment. (P 9)

The participants attended upper secondary school. One obstacle for dental visits was schoolwork; another was that some participants had jobs after school. Also, going to school was often judged as more important than going to the dental clinic. How schoolwork was prioritized in relation to dental care differed from individual to individual, with some participants prioritizing school while others said they wanted to visit the dental clinic during school time. To have an appointment early in the morning, i.e., before school started, was described as feeling like an extra school lesson, which was not very popular. In the morning before school, the participants preferred to sleep; after school, they preferred doing something else. Therefore, both the time of the dental appointment and the length of the visit was important for the participants’ motivation to keep their appointment at the dental clinic.

If you’re still at work or at school and you have an appointment, then maybe it’s important that you … that it doesn’t take too long considering that you might miss much in school. (P 11)

#### Transportation difficulties

Transport issues in getting to the dental clinic were described among those participants who did not have a driver’s licence. Some described that they had to take the bus or even take several different buses to get to the clinic. Usually those participants lived in one area, went to school in another, while the dental clinic was located in a third area. Despite such difficulties, these participants kept their dental appointments most of the time because they perceived them as important.

Because I need to take the bus from [name of the neighbourhood] into the city and then change from the city to [name of another area]. But, as I say, I do it. I still think it’s important to go to the dentist. (P 3)

#### Difficulties getting in contact

Participants expressed frustration about getting in contact with the dental clinic, often in the context of wanting to cancel their dental appointments by phone. They preferred talking to someone at the dental clinic to just being referred to cancel or reschedule the appointment on the Internet. Sometimes they were placed in a phone queue, which caused them stress. The participants were also worried that they would have to pay for late cancellation if they were unable to get through to the clinic.

So I had to book an appointment there and then … I had to be in a phone queue again, quickly. Because if I didn’t cancel the appointment a day before I would have to pay the fee but she said that I could get 1 hour to make a cancelation (P 5)

### Free of charge

The participants appreciated the fact that dental care for all children and adolescents in Sweden is free of charge. They were aware that children and adolescents in other countries do not have access to free dental care. The two subcategories Make sure to go for dental care while it’s free and Wouldn’t pay for it constituted this category.

#### Make sure to go for dental care while it’s free

Dental care was mostly described as a service you should use when you do not have to pay for it. Many participants thought that because dental care is free of charge more adolescents attend their appointments than would be the case if they had to pay the dentist.

I think it [free dental care] is fantastic. It makes people go there. Many adolescents don’t have a lot of money and then the dental visit is not something they choose to prioritize, which means that you won’t go there. But if it’s free and you get an appointment by letter and … (P 9)

Free dental care for young people gave the participants an opportunity to maintain regular dental visits. Moreover, free dental care also made it possible for them to seek help when they had problems with their teeth. They said that the opportunity to have regular dental visits and get help with their teeth gave them a better chance of having nice and healthy teeth.

No, oral health is important and I want to be able to get the chance to feel satisfied with my teeth and if it had cost money then I think I may not have had that opportunity. (P 4)

When talking about dental health later in life, the participants expected that they would develop caries disease or that they would not take proper care of their teeth in the future. Several expressed that they would probably not be able to pay for dental visits because of high costs.

#### Wouldn’t pay for it

Even though going to the dental clinic was seen as necessary and important, the participants were doubtful that they would continue going to the dental care once the visits were no longer free of charge. Thinking about themselves as young adults, they said that they were unsure what their financial situation would be. They also talked about moving away from home into their own place and said they would prefer saving the money instead of paying for dental care. They would rather pay for other things, such as travel, clothes and food, than for dental check-ups.

You have no regular income and if you have to pay for dental visits, you may choose not to go. Even if it’s important for adolescents because it determines how you will look and what your teeth will be like when you get older and how long you can manage, and so on. So you may choose not to go to the dentist just because it costs money. And you might want to spend money on something else. (P 5)

## Discussion

In search for a deeper understanding of experiences of dental care, this study and its findings could be summed into the theme “Triggers for adolescent girls to take on or not take on adult responsibility for dental care” and further summarized in five main categories: Pain and discomfort; Attractive and healthy teeth; Feeling safe and secure; Taking on the responsibility; and Free of charge.

Despite the fact that the girls in the present study had missed dental appointments in the past, they expressed the importance of dental care. However, they prioritized going to doctor, going to school, working, and being with friends over attending their dental appointments. This may not be surprising as previous studies reported similar results (Östberg et al., 2010; Skaret et al., 2000).

The present study revealed several conditions that could facilitate or hinder dental attendance. During the interviews, the participants frequently stated that knowing what will happen at the dental clinic was the most important aspect that could affect whether they would go to the dental clinic. A feeling of safety when visiting dental clinic could be facilitated in several ways. For instance, the continuity of seeing the same dental personnel was, according to the interviewed girls, important in helping them develop trust and feel secure during the visit to the dentist. These findings were also revealed in a Swedish study (Hedman, Gabre, Birkhed, & Lepp, 2013) showing that having the same dental hygienist helped both adolescent girls and boys develop trust. However, in the present study, seeing the same dental personnel was not always enough to feel safe and secure. Having a parent or friend accompany them to the dental clinic was described as having a positive impact on their dental visits.

The girls in the present study expressed several aspects that could be seen as barriers to accessing dental care. We know from previous studies that negative dental experiences during childhood can affect dental visits later in life. This may be due to painful and unpleasant dental experiences that can develop into dental anxiety (Levin, Proter, & Levin, 2007; Skaret et al., 2007, 2000) and, in turn, lead to irregular dental attendance (Fägerstad et al., 2018; Hattne, Folke, & Twetman, 2007; Vika et al., 2006). Also, regarding dental personnel as strangers, as well as having to keep the mouth open for a long time made these girls feel vulnerable and exposed. Even though the participants in our study were 16**–**19 years old, these findings correspond to another Swedish study showing that having to open the mouth for strangers during a dental treatment could make 15-year-old boys and girls feel exposed (Bergström, Sköld, Birkhed, & Lepp, 2012). Other aspects that could be seen as barriers to dental visits were being treated roughly by the dentist and having sharp dental instruments put in the mouth, which in turn could be associated with pain (Dodd et al., 2014).

Finally, the uncertainty of who is responsible for the adolescents’ dental visits needs to be discussed. Some participants stated that their parents were responsible for taking them to the dentist while others expressed that going to the dentist was their own responsibility. No matter whose responsibility dental visits were, the participants in the present study still wanted their parents to have contact with the dental care. The degree of responsibility for own dental care may depend on adolescents’ developmental stage during the transition period between adolescence and adulthood. What we need to keep in mind is that adolescents’ autonomy is not the same as independence from their parents.

The ability of individuals to make their own decisions and manage on their own improves greatly during adolescence (Hwang, 2018). Additionally, when children enter adolescence, their parents are still largely responsible for all aspects of their health. By the time these individuals reach the end of adolescence, they have almost entire responsibility for their health (Christie & Viner, 2005).

What facilitated or hindered participants in the present study from attending their dental appointments may be illustrated by aspects of Self-Determination Theory (SDT) (Deci & Ryan, 1985). According to SDT, we have innate psychological needs that are the basis for self-motivation, while we tend to seek situations that satisfy our needs. One of these innate needs is competence and several participants in the present study expressed that it was important for them to know what will happen in the dental clinic. Dental personnel who let their patients be involved in their own dental treatment may contribute to increased perceived competence and feelings of capability of managing to handle a potentially unpleasant dental visit. In the present study, another factor that facilitated regular dental attendance was having personal continuity of care, which helped many of the participants go to the dental clinic. This aspect could be seen as an example of relatedness, which is another of the needs described by SDT. The third need identified by SDT is autonomy and, as described above, the need to feel agency and to do things that are coherent with one’s own values is a theme that emerged in the interviews. The wish to take on increasing responsibility for one’s own dental health can be understood as a need for autonomy.

Although we cannot validate SDT as we are not testing this theory in the present material, it may help us to create an SDT-based intervention that can help adolescents to attend their dental appointments by focusing on their psychological as well as dental needs.

### Methodological considerations

To ensure trustworthiness of this study, concepts such as transferability, credibility, dependability and confirmability were used (Graneheim et al., 2017). One aspect of credibility is to include participants who have experienced the phenomenon under study and who can talk about it. We conducted purposeful sampling to achieve diversity of participant age and gender and PDC location in areas with different sociodemographic profiles in order to obtain broad data. Further, we have explained and described the selection of the participants, data collection and analysis in detail, allowing readers to determine whether our results are transferable to other contexts.

The study findings have provided some insight into how adolescent girls with low rates of missed dental appointments experience dental care. Our intention was to interview both girls and boys 16**–**19 years of age who repeatedly missed their dental appointments. This objective proved to be challenging and we failed to get in contact with boys in general and also with adolescents who had missed their dental appointment repeatedly over a long period. Therefore, we may have missed some aspects of the phenomenon under study. During the recruitment period, we only managed to get in contact with one boy and 17 girls with at least one missed dental appointment according to the dental records. Moreover, the boy and one girl did not show up at the interview, and four girls declined to participate. Considering the fact that missed dental appointments are more common among boys than among girls (Östberg et al., 2010; Skaret et al., 2007, 1998, 2000), having boys participating in the study would probably have given a wider perspective, and may have revealed gender variations regarding experiences of dental care.

Another aspect that could be considered a limitation in this study is the lack of information regarding the history of missed dental appointments among the participants. Secondly, though we have stated that the participants were enrolled in upper secondary school, further information about the type of upper secondary education is missing. These aspects may reduce the credibility of the study results.

However, although only girls agreed to participate and the heterogeneity of the group of participants was therefore limited, the present study can still be considered to increase the knowledge about adolescent girls’ experiences of dental care in a Swedish context.

To ensure the dependability of the study, we used semi-structured interviews and described the questions in the interview guide. What may be worth mentioning is that the first author who carried out the interviews, is a registered dental hygienist with a pre-understanding of free dental care for children and adolescents. This may have had an impact on the interviews and data analysis. However, the first author tried to put the pre-understanding aside by being as objective as possible in order to analyse and interpret the results as transparently as possible. To increase the study’s dependability, the analysis was performed together with one of the co-authors (E.C.), who is a registered nurse. The other co-authors (K.A. and J.L.) had a validating role through the analysis process by discussing each category and subcategory, as well as the main theme, to reach consensus. Further, two independent researchers reviewed the study design, analysis and findings, which is important in terms of confirmability.

Four girls were invited to participate in a telephone interview because they had difficulties in finding time for a face-to-face interview. Interviewing adolescent girls by telephone was experienced as challenging as there was no more interaction between the participants and the interviewer than their statements and voices (i.e., body language was missing) (Musselwhite, Cuff, McGregor, & King, 2007). This may have affected the length of these interviews, which were shorter than face-to-face interviews.

## Future research and clinical implications

The results of this study provide an insight into how adolescent girls with low rates of missed dental appointments experience dental care in a Swedish context. Future research should focus on both genders and on adolescents’ experiences of dental care in a Swedish context in order to get a broader picture of the studied phenomenon. It would also be desirable to include adolescents with a consistent pattern of missed or cancelled appointments, as we did not manage to get in contact with them in the present study. One possible way of getting in contact with these individuals may be to recruit them in the schools. Another alternative for recruiting adolescent participants would be to advertise future research via the social media (Amon, Campbell, Hawke, & Steinbeck, 2014; Whitaker, Stevelink, & Fear, 2017). Further, using online focus groups (Boateng et al., 2016; Tates et al., 2009) among adolescents may be a preferable way of data collection from participants that are hard to reach using the traditional methods.

Since the girls in this study frequently expressed the need to feel safe and secure, it would be interesting to examine dental personnel’s perceptions regarding meeting and communicating with adolescent dental patients. In this context, we need to consider whether SDT-based interventions developed for dental attendance may be suitable.

Also, considering the results of this study, and particularly regarding the importance of feeling safe and secure, we may need to provide adolescent dental patients with more information and involvement when planning for their dental visits and treatments. Therefore, we need to ensure that adolescents understand what will happen during their dental visits. Further, we should take all efforts to avoid discomfort and painful treatments as far as possible since negative dental experiences may be seen as risks for irregular dental attendance.

## Conclusions

In conclusion, this study describes the experiences of dental care of adolescent girls with missed dental appointments, in a Swedish context. Based on the results of this study, several potential conditions could be seen as barriers and facilitators to accessing dental care. These aspects should increase the knowledge on how to meet and treat adolescent girls in dental care. Knowing what will happen during the dental visit was highlighted by the participants as decisive to whether or not they would attend their dental appointments. With this in mind, we should as far as possible ensure that our patients feel safe and secure by making them involved in their dental visits and by trying to avoid painful treatments.
